# Supplementary material for: The effect of virtual visual scene inclination transitions on gait modulation in healthy older versus young adults—A virtual reality study
Source: PLoS One. 2024 Dec 4;19(12):e0311315. doi: 10.1371/journal.pone.0311315 (PMC11616833; doi:10.1371/journal.pone.0311315)
Supplement: S1 File — Additional explanatory information to support the methods (sections A-B) and discussion (section C) of this study. (DOCX) [file pone.0311315.s001.docx]

1. **Supporting information for the *Methods* section, subsection *Apparatus*:**

VR version of the Rod and Frame Test

The rod-and-frame test was used to assess the visual field dependence for each participant. The test measures how visual perception of the orientation of a rod is influenced by the orientation of a peripheral frame around it. The test was implemented in our lab using Unity software and C# scripting. The participants sat straight in front of a computer screen wearing head mount device (HMD) VR glasses (HTC VIVE, HTC; New Taipei City, Taiwan). They were instructed not to tilt their head or move during the test. The VR environment included a central white rod (11° long) with a peripheral frame around it (occupying ~16X16° of the visual filed) rotated at a trial-specific orientation. The center of the rod and the frame were aligned, but each one with its own independent orientation. Both the rod and the frame were white and presented on a black background (screen resolution was 1920X1080). During the test, a sequence of 28 trials were consecutively presented during which the frame was tilted at one of seven possible random positions: 0/±10/±20/±30 degrees (0 was vertical, + was clockwise), each position was presented four times(1). For each trial, the rod was at a random orientation (sampled from 0-180 degrees range distribution), regardless of the position of the frame. The participants’ task was to orient the rod upright (i.e., perpendicular) to the true horizon, irrespective of the surrounding frame’s orientation. This was achieved by rotating the rod around its center in either direction using the VR system’s remote control, the surrounding frame was not changed by this manipulation. Once the participants estimated it as being upright, they pressed a button on the remote control, which led to the clearing of the display and the beginning of another trial.

1. **Supporting information for the *Methods* section, subsection *Procedure*:**

Steady-state velocity

A real-time algorithm monitoring treadmill speed determined the SSV. According to the algorithm, SSV is attained after (i) minimum 30s of walking from the beginning of the trial, and (ii) a consecutive period of 12s with gait speed coefficient of variance less than 2%. Upon satisfying both conditions, the transition of the treadmill and/or visual scene inclination (as appropriate for the experimental condition) was automatically triggered.

**C. Supporting information for the *Discussion* section:**

*Double incongruent walking conditions*

When the treadmill transitioned upwards (c.f. figure 2, upper row), both groups showed a steady decrease in walking speed across the whole trial, and as expected the older adults walked slower than the young group. Interestingly, when the treadmill transitioned downward both groups showed different walking patterns (c.f. figure 2, lower row). In the congruent T_D_V_D_ condition, while the young group increased their gait speed, the older adults initially applied the braking effect, possibly until they felt in control, and only then let the gravity force accelerate their speed. The deterioration of the multi-sensory integration with aging is seen nicely when the treadmill transitioned downward, and the visual scene remained either leveled or transitioned upward. It appears that an initial increase in gait speed is followed by a decrease in gait speed, and only after about 20 seconds, a steady gait speed is reached. We suggest that the phenomenon is seen primarily when walking downwards, because of an a priori perception that walking upward is less ‘frightening’ and the physical restrictions seen with aging govern on the discrepancy in perception, which eventually is causing a decrease in walking speed. In contrast, while walking down one fears to lose control and fall so the multi-sensory integration is more tuned to maintain stability.

**References for the supplementary file**

1. Bagust J. Assessment of verticality perception by a rod-and-frame test: Preliminary observations on the use of a computer monitor and video eye glasses. Arch Phys Med Rehabil. 2005;86(5):1062–4.
