# Supplementary material for: The effect of virtual visual scene inclination transitions on gait modulation in healthy older versus young adults—A virtual reality study
Source: PLoS One. 2024 Dec 4;19(12):e0311315. doi: 10.1371/journal.pone.0311315 (PMC11616833; doi:10.1371/journal.pone.0311315)
Supplement: Attachment — Submitted filename: Response to Reviewers_Benady.docx [file pone.0311315.s002.docx]

**Response to the Editors and Reviewers**

We thank the editorial board members and the reviewers for their useful comments. We have addressed each and every one of them and incorporated needed revisions in the revised manuscript*.* The feedback from the reviewers was particularly helpful for strengthening our statistical analyses, description of the methodology and general readability throughout the revised manuscript and also, in clarifying several issues. Point-by-point response to each of the comments of the reviewers is provided below .

*Response to comments made by reviewer #1:*

***Comment1:*** *“What is the impact of visual acuity and sensorimotor processing on the visual field dependency in older adults? Discussing the relevant literature may help provide additional context for the stated hypotheses.”*

**Response:** We thank the reviewer for pointing this out. In the revised introduction we now elaborate on the possible interaction between changes in sensorimotor processing and increased reliance on visual cues (visual dependency) in older age. With respect to visual acuity, interestingly, although known to weaken with age, we found no evidence supporting vision acuity as an influencing factor which changes or diminishes the compensatory mechanism of increased visual dependency with age (p.5, par.1).

*"…This reduction in older age is due to several changes that occur with aging and involves alterations in the MSI component weights i.e., delayed and/or decreased vestibular and somatosensory influx, which in turn increase visual dependency with aging [14-16]. Interestingly, this compensatory increase of visual dependency in the MSI takes place even though visual acuity is known to deteriorate with age [17]. "*

***Comment2:*** *“Was there an a-priori justification for the sample size selected? If not, please be sure to include effect sizes for all outcome measures to evaluate if study was sufficiently powered to detect changes.*

**Response:** Thank you for raising this important issue. We now report effect size along with our revised statistical analysis results, using the rank biserial correlation, in accordance with the non-parametric approach of the Man-Whitney test which was selected following comment #6 of reviewer #2 (p.13, par.1; p.13-14,16 throughout the revised results section).

*"… and results effect size was estimated using the rank biserial correlation (r_rb_), in accordance with the non-parametric approach."*

***Comment3:*** *“How were physical and visual restrictions assessed? Were there visual testing performed, or were self-reported evaluations, or medical records used? Was color blindness assessed? Was visual acuity assessed, or need for bifocals, or corrective lenses?”*

**Response:** Thank you for this comment which motivated us to elaborate, in the revised methods section, in what manner we evaluated physical and visual restrictions of potential participants. Before entering the study, participants were required to self-report having vision acuity 20/20 (or corrected to 20/20 by means of glasses/contact lenses) and no color blindness. Regarding their physical state, we specifically inquired about any neurological, vestibular or orthopedic conditions which may hinder their ability to walk or if there are any other movement restrictions (which were all considered as exclusion criteria). To ascertain inclusion criteria, at the beginning of the experimental session, in the habituation period, the ability of the participant to walk was verified by the examiner and subjects were asked to confirm detecting the trail and parts of the scenery of the visual arena (p.6, par. 1).

*"**Specifically, participants were required to self-report having vision acuity 20/20 (or corrected to 20/20 by means of glasses/contact lenses) and no color blindness. Additionally, participants were inquired about any neurological, vestibular or orthopedic conditions that may hinder their ability to walk, in addition to any other movement restrictions."*

***Comment4:*** *“Did the authors consider controlling for multiple comparisons, given large set of analyses?*

**Response:** Following the reviewer's counsel we consider the significance level ("Alpha") of our statistical analysis to be 0.025 instead of 0.05, now accounting for two outcome measures (peak amplitude and timing) (p.13, par.1).

*"p-value of equal or less than 0.025 (considering two variables) indicated a significant difference."*

***Comment5:*** *"* *Results would benefit from reporting of effect sizes in test.*

**Response:** We agree with the reviewer, please see response to comment #2 above. We now include in the revised result section of the manuscript the rank biserial correlation for each statistical comparison reported (p.13-14,16 Throughout the revised results section)

***Comment6:*** *"* *Be sure to use higher resolution or vectorized figures, as the current figures were difficult to read.”*

**Response:** Following the reviewer's suggestion, we replaced Figure 1, which was indeed in a sub-optimal resolution and reconfirmed that we are adhering to the specific formatting requirements for figures provided by the journal.

***Comment7:*** *"* *Good discussion of the findings in relation to relevant literature.”*

**Response:** We thank the reviewer for this encouraging feedback.

*Response to comments made by reviewer #2:*

***Comment1:*** *" Authors have used the abbreviation “IMG” without introducing it. Furthermore, I think that it could be a typo and they meant MSI instead."*

**Response:** We thank the reviewer for noticing this inconsistency. Indeed, the authors intention, in the two instances where IMG was used, was for the abbreviation MSI (Multi- Sensory Integration). We have now corrected the manuscript in these two locations. (p.11, par.2; p.18, par.2).

***Comment2:*** *" A positive ratio indicates that both WS and a free body accelerate/decelerate in the same direction (i.e., both accelerate when downhill and decelerate when uphill). A ratio further from zero suggests more gravitational influence on walking.” Could the author here confirm if all the values will be positive or if there is an unmentioned interpretation of negative values?"*

**Response:** According to equation 1 the aforementioned ratio is the outcome of dividing the area under the curve of WS by the area under the curve of the velocity of a free body. The sign is defined in accordance with the direction of the velocity change (acceleration/deceleration). Thus, the ratio can indeed be negative if WS change is in an opposite direction to this of the velocity of a free body. An example of the conditions leading to a negative ratio can be found in the older adults group for downhill walking. While a free body in a downhill scenario accelerates uncontrollably, the older adults group demonstrated WS decrease (deceleration), peaking below the SSV. This will create a negative ratio as depicted in figure 4 (upper panel; orange dots). No unmentioned interpretation nor adjustments to negative results were carried out in this section. We include the next clarification in the revised methods section (p.10, par.4).

*"A positive ratio indicates that both WS and a free body accelerate/decelerate in the same direction (i.e., both accelerate when downhill and decelerate when uphill). A negative ratio indicates that WS acceleration was in the opposite direction from this of a free body. A ratio further from zero suggests more gravitational influence on walking."*

***Comment3:*** *"* *Equations 4 and 5 requires further clarification in describing the rationale behind and defining all the terms giving attention that I observed the usage of Wvisual-cues and Wvision while the difference between them is not clear. The same applies to Wbody-based and Wbody-based cues."*

**Response:** We thank the reviewer for raising the unclarity regarding the formulations of equations 4 and 5. We adjusted the terminology of model parameters to be more coherent and easier to follow and further revised the descriptions in this part of the revised methods section (p.11, par. 2).

"*Given that locomotion is maintained by MSI, we used a linear weighted summation model [1] to estimate the weight of visual and body-based cues [4]. The general model is presented by the following equation:*

$${WS}_{combined}= W_{visual cues}*{WS}_{vision driven}+W_{body-based cues}*{WS}_{body-based driven} (2)$$

*WS_combined_ is defined as the resulting integrated behavior after summing the relative part of each component. On the right side of the equation, W denotes the weight of a unimodal cue (visual or body-based) and WS refers to the walking speed which was driven solely by that cue (visual or body-based). We assume that WS in conditions T_L_V_U_ (WS_vision driven, up_) and T_L_V_D_ (WS_vision driven, down_) are driven exclusively by uphill and downhill visual cues, respectively. Contrariwise, we assume that WS in conditions T_U_V_L_ (WS_body-based drivan, up_) and T_D_V_L_ (WS WS_body-based drivan, down_) are driven exclusively by the corresponding body-based cues. While assuming that vision and body-based cues are the main factors influencing locomotion, the weight of each unimodal cue can be calculated using the following general equations:*

$$W_{visual cues}+W_{body-based cues}=1 (3)$$

$$W_{visual cues}=\frac{\left( {WS}_{combined}-{WS}_{body-based driven} \right)}{\left( {WS}_{vision driven}-{WS}_{body-based driven} \right)} \left( 4 \right)$$

$$W_{body-based cues}=\frac{\left( {WS}_{combined}-{WS}_{vision driven} \right)}{\left( {WS}_{body-based driven}-{WS}_{vision driven} \right)} (5)$$

*recall WS_combined_ represents the congruent conditions T_U_V_U_ and T_D_V_D_ (i.e., combined cues) and can be calculated using the following equations for each respective inclination:*

$${WS}_{conbined, up}= W_{vision cues,up}*{WS}_{vision driven,up}+W_{body-based cues,up}*{WS}_{body-based driven,up} (6)$$

${WS}_{conbined, down}= W_{vision cues,down}*{WS}_{vision driven,down}+W_{body-based cues,down}*{WS}_{body-based driven,down}$*(7)*

*Notably, to avoid repetition of mathematical factors ('using data to explain related data'), the predicted behavior of uphill and downhill walking was estimated using the opposite unimodal weights i.e., both visual and body-based weights derived from downhill conditions was used for the prediction of uphill walking, and the uphill derived weights was used for downhill walking."*

***Comment4:*** *"* *Statistical analyses Values are presented by their group mean values (± SE).” The term values should be more specific in referring to which values."*

**Response:** We thank the reviewer for this comment. Our intention was to the values described in the following paragraph. In the revised version we omitted this sentence to avoid future confusion and kept this statement in the introducing paragraph of the results section. (p.13, par. 2).

***Comment5:*** *"* *The after effect, comparing the time of return from the peak/trough…”. The after effect term is not so clarifying."*

**Response:** We thank the reviewer for this comment. The following clarification is now part of the 'statistical analysis' subsection of the revised methods section (p.13, par.1).

*"Additionally, we calculated and compared the time it took the participant to return from the peak/trough velocity to SSV."*

***Comment6:*** *"Statistical analysis were done using t-test to compare the results, were the data tested for normality?."*

**Response:** We thank the reviewer for raising this important issue. Indeed, the majority of data sets included in the statistical analysis of this study were normally distributed. However, in the older adults group several outcome measures of the time to peak, were not. Accordingly, and to assure robust statistical assessment in the revised manuscript we choose a non-parametric approach using the Man-Whitney test for group comparisons and the Spearman's correlation coefficients were computed to evaluate associations between outcome measures. The following citations were included in relevant location of the revised methods and results sections and all required correction were implemented (p.12, par.2).

*"In pre-hoc analyses, Shapiro-Wilk normality tests were conducted on the time and amplitude of peak outcome measures (in both T_L_V_U_ and T_L_V_D_ conditions), for each age group (n=2; young and older adults). Of the eight data sets assessed, normality tests indicated partially non-normal distributions for the older adults group. Accordingly, a non-parametric approach was chosen for the post-hoc age group comparison using the Mann-Whitney test. We than confirmed all outcome measures sphericity (i.e., equal variances of the differences between all groups) using the Levene's test (p>0.05)."*

*"…Spearman correlation coefficient was computed to evaluate the association between visual field dependence index and (i) the standardized response to virtual inclination and (ii) the age of participants."*

***Comment7:*** *"Authors have to add more description of the results from Fig2 regarding the cases where the Treadmill was not Ground levelled in the section of Gait speed modulations following physical and/or virtual inclination transitions since in the current version it included only information of conditions where treadmill was in ground level mode".*

**Response:** We thank the reviewer for this comment which driven us to include explanations regarding the non-level inclination results in the main document instead of the supplementary material file to strength the inclusiveness of the results section. The following is now brought in (p.14, par. 2).

*"For the treadmill up conditions there was a gradual monotonic change in speed in both groups, with a more prominent effect in the older adult group (see Figure 2, first row, purple line (older adult group) is lower (i.e., slower) than the orange line (young group)). Interestingly, in the treadmill down conditions both groups showed opposite walking patterns; in the congruent condition T_D_V_D_, while the young group constantly increased their walking speed, the older adult group initially braked and decreased their speed, with a monotonic increase during the next minute after the transition. This pattern was roughly the same also in the T_D_V_U_ and the T_D_V_L_ conditions."*

***Comment8:*** *"In the first part of the discussion, there is a repetition of the information regarding the results of the differences in the magnitude and timing of the braking and exertion effects between young and elderly group, however it needs to be further discussed and interpreted in expansion with respect to the results section."*

**Response:** Thank you for this comment. Our intention in this initial paragraph was to summarize the study results to increase readability of the following discussion sections in which more in-depth explanations and interpretations are included. We accept the reviewer's comment suggesting this part to be partially redundant and in the revised manuscript we shorthand it.

***Comment9:*** *"Herein we strengthen the notion that visual field dependency increases with aging [26-27], this can be seen by (1) significant increase in the older adults group compared to the young group” Increase in what? In the visual field dependency?"*

**Response:** Thank you for this comment. Our intention was indeed to describe the increased visual dependency of the older adults group compared to the young, which is reflected both through a higher rod and frame index and by a positive correlation to age within this age group. We rephrased the explanation in this section in the revised manuscript (p.20, par. 2).

*"Herein we strengthen the notion that visual field dependency increases with aging [26-27], this can be seen by (1) significant increase of visual field dependency as reflected by the 'road and frame' test index, in the older adults group compared to the young group, and (2) only within the older adults, there is a significant positive correlation between age and rod and frame index."*

***Comment10:*** *"Clinical values of the findings should be more emphasized within the discussions."*

**Response:** We thank the reviewer for this suggestion which we implement in the revised discussion section. The clinical values of this study's findings are now emphasized and delivered in a more concise and clear way. (p.22, par.1).

*"Overall, senses and body functions are known to deteriorate with aging, such as vision [35], joint mobility [36], muscle force [37] and balance [16]. However, age-related changes of multi-sensory integration are less studied. The changes in performance of MSI tasks may be more sensitive and aid as an earlier predictor to detect future deterioration in daily living activities compared to assessing each sense by itself. Taking a more holistic approach in which you evaluate the integrated outcome of sensorimotor function instead of its uni-sensory inputs can be potentially beneficial in the clinical arena where diagnostic tools and early markers could be developed based on performance of daily MSI tasks."*

**References added:**

[17]	Freeman EE, Egleston BL, West SK, Bandeen-Roche K, Rubin G. Visual acuity change and mortality in older adults. Investig Ophthalmol Vis Sci 2005;46:4040–5.
